# Perioperative prevention of venous thrombosis with low-molecular-weight heparin versus rivaroxaban in elderly patients with hip fractures: A retrospective controlled study

**DOI:** 10.1097/MD.0000000000047339

**Published:** 2026-01-30

**Authors:** Xueqin He, Hui Chen, Fan Lou, Chen Yan, Jing Feng

**Affiliations:** a Clinical Pharmacy Department (Pharmacy Section), West China Hospital of Sichuan University, Chengdu City, Sichuan Province, China.

**Keywords:** anticoagulation, elderly patients, hip fracture, low-molecular-weight heparin, rivaroxaban, venous thrombosis

## Abstract

Elderly patients with hip fractures are at significantly increased risk of perioperative venous thromboembolism due to multiple comorbidities, functional decline, and prolonged immobilization. Balancing effective thromboprophylaxis with minimizing bleeding risk and improving adherence remains a core challenge in clinical management. Low-molecular-weight heparin (LMWH) is the conventional prophylactic agent, but its injectable administration often results in poor compliance. Rivaroxaban, an oral factor Xa inhibitor, has shown promise; however, clinical evidence in elderly populations remains insufficient. This study aimed to compare the efficacy and safety of rivaroxaban versus LMWH in elderly patients with hip fractures during the perioperative period. A total of 100 elderly hip fracture patients admitted between January 2022 and January 2025 were enrolled and assigned to either the LMWH group (n = 50) or the rivaroxaban group (n = 50). Baseline characteristics, including age, sex, body mass index, blood pressure, heart rate, comorbidities, and surgical methods, showed no significant differences between groups (*P* > .05). Perioperative outcomes compared included incidence of venous thrombosis, bleeding complications, mortality, medication adherence, and postoperative recovery. The incidence of deep vein thrombosis was significantly lower in the rivaroxaban group compared with the LMWH group (4.0% vs 10.0%, *P* = .03), with a higher thrombosis-free survival rate confirmed by Kaplan–Meier analysis. The overall bleeding event rate was comparable (8.0% vs 6.0%, *P* = .75), with no severe bleeding in either group. Thirty-day mortality was low in both groups without significant difference (4.0% vs 2.0%, *P* = .69). Rivaroxaban demonstrated slightly better adherence (nonadherence rate 2.0% vs 6.0%, *P* = .12). Postoperative recovery was more favorable in the rivaroxaban group, with shorter hospital stay (9.2 ± 2.3 vs 10.1 ± 2.5 days, *P* = .04) and earlier 1st ambulation (3.8 ± 1.2 vs 4.4 ± 1.3 days, *P* = .036), while wound healing time did not differ significantly. Rivaroxaban provides superior antithrombotic efficacy compared with LMWH in elderly hip fracture patients during the perioperative period, with comparable safety, better treatment adherence, and enhanced postoperative recovery. These findings suggest rivaroxaban may represent a preferred prophylactic option in this high-risk population, although larger multicenter trials with longer follow-up are warranted to validate its clinical utility.

## 1. Introduction

Hip fractures are one of the most common and serious orthopedic injuries among the elderly, and their incidence continues to rise with the acceleration of global population aging.^[1,2]^ Epidemiological investigations have shown that hip fractures not only lead to high disability rates and functional impairment but are also closely associated with increased mortality, making it a major public health problem that severely impacts both the quality of life of elderly patients and the healthcare system burden.^[3,4]^ Elderly individuals often present with multiple comorbidities and diminished physiological reserve, resulting in substantially elevated perioperative complication risks. Among these, venous thromboembolism (VTE) is particularly common, with deep vein thrombosis (DVT) and pulmonary embolism posing direct threats to patient survival.^[5,6]^ Thus, balancing effective thromboprophylaxis with minimizing bleeding risk remains a critical challenge in the perioperative management of elderly hip fracture patients.

In clinical practice, low-molecular-weight heparin (LMWH) has long been the standard prophylactic anticoagulant due to its well-defined mechanism, favorable safety profile, and established efficacy.^[7,8]^ Administered subcutaneously, LMWH has a rapid onset of action, a short half-life, and minimal drug interactions, with abundant evidence supporting its effectiveness.^[9,10]^ However, LMWH is not without limitations. The injectable route can be complex and inconvenient for elderly patients and caregivers, often leading to poor compliance. Additionally, impaired renal function – frequently present in elderly patients – may affect drug metabolism and increase bleeding risk. Long-term injection further adds to the caregiving burden and negatively affects patient comfort and quality of life.^[11–13]^ With advances in anticoagulation strategies, there is a growing demand for alternative agents that are safer, more convenient, and better suited for elderly populations.

Rivaroxaban, an oral factor Xa inhibitor, has emerged as a promising alternative in perioperative anticoagulation management.^[14,15]^ Its advantages include oral administration, fixed dosing, and the absence of routine monitoring requirements, and it has been widely applied in hip and knee arthroplasty.^[16,17]^ Multiple clinical trials have demonstrated its efficacy in reducing thrombotic risk, without a significant increase in major bleeding events.^[18–20]^ Nevertheless, most of these studies have focused on elective joint replacement patients. In contrast, elderly patients with hip fractures present with acute onset, multiple comorbidities, and higher perioperative risks, making their response to anticoagulation therapy potentially different. Current evidence in this high-risk population remains limited, particularly regarding the comprehensive evaluation of efficacy, safety, and adherence.

Preliminary studies suggest rivaroxaban may reduce the incidence of DVT compared with LMWH while offering improved compliance due to its oral formulation.^[21,22]^ However, most available studies suffer from small sample sizes and limited endpoints, focusing mainly on thrombosis and bleeding outcomes. Little attention has been paid to other clinically relevant outcomes, such as adherence, length of hospitalization, and postoperative recovery. For elderly patients, treatment adherence and recovery speed are directly linked to quality of life and healthcare resource utilization, yet these important clinical outcomes remain underexplored.^[23,24]^ Therefore, comparative evaluation of rivaroxaban and LMWH in VTE prevention, bleeding risk, adherence, and recovery among elderly hip fracture patients holds both theoretical and practical significance.

Against this background, the present study focused specifically on elderly patients with hip fractures and employed a retrospective controlled design to compare rivaroxaban and LMWH during the perioperative period. In addition to assessing VTE prevention efficacy and safety, the study incorporated adherence and postoperative recovery outcomes, thus providing a multidimensional evaluation of both agents.

## 2. Method

### 2.1. Study population

This study was approved by the Ethics Committee of West China Hospital. This was a single-center, retrospective controlled study. A total of 100 elderly patients with hip fractures who underwent surgical treatment in our hospital between January 2022 and January 2025 were enrolled. Inclusion criteria were: age ≥65 years; definitive diagnosis of femoral neck fracture or intertrochanteric femoral fracture; treatment with hemiarthroplasty, total hip arthroplasty, or proximal femoral nail antirotation fixation; and availability of complete clinical records and follow-up data. Exclusion criteria included: presence of malignant tumors, coagulation disorders, or contraindications to anticoagulation therapy, as well as perioperative loss to follow-up or incomplete data. The sample size was determined based on preliminary experimental data and calculated using G*Power 3.1 software (Heinrich Heine University Düsseldorf, Düsseldorf, North Rhine-Westphalia, Germany).

### 2.2. Groups and interventions

All patients were assigned, according to different treatment methods, to either the LMWH group (n = 50) or the rivaroxaban group (n = 50). Patients in the LMWH group received subcutaneous injections of sodium LMWH starting 24 hours after surgery at standard prophylactic doses, which were continued post-discharge for 2 to 4 weeks. Patients in the rivaroxaban group were administered rivaroxaban orally at a dose of 10 mg once daily, beginning 6 to 10 hours after surgery and continuing for 2 to 4 weeks. Both groups also received standard perioperative management, including prophylactic antibiotics, fluid replacement, analgesia, and routine rehabilitation care.

### 2.3. Outcome measures

Baseline clinical characteristics were recorded, including age, sex, height, weight, body mass index, blood pressure, heart rate, smoking history, comorbidities (hypertension, diabetes mellitus, coronary heart disease, chronic obstructive pulmonary disease, etc), and surgical procedure.

The primary outcome was the incidence of perioperative DVT, categorized into proximal and distal thrombosis, with the time of onset documented. Secondary outcomes included the incidence of bleeding complications (classified as mild subcutaneous ecchymosis, wound bleeding, gastrointestinal bleeding, or major bleeding events), overall mortality and 30-day cumulative survival, medication adherence with causes for nonadherence, and postoperative recovery indicators (length of hospital stay, time to 1st ambulation, and wound healing time).

### 2.4. Follow-up and assessment

All patients underwent continuous monitoring during hospitalization and were followed up for 4 to 6 weeks after discharge. Follow-up was conducted through outpatient visits and telephone interviews to ensure data completeness and to evaluate treatment adherence.

#### 2.4.1. Evaluation of DVT

All patients underwent routine lower limb color Doppler ultrasonography on postoperative day 7, day 14, and before discharge, with additional assessments performed if clinically indicated. Patients presenting with leg swelling, pain, or superficial vein engorgement underwent immediate repeat ultrasonography for confirmation. DVT was classified as proximal or distal, and diagnostic criteria were based on the presence of mural thrombus within the vessel lumen or evidence of obstructed blood flow on vascular ultrasound.

#### 2.4.2. Evaluation of bleeding complications

All bleeding events were documented by attending physicians and graded according to the International Society on Thrombosis and Haemostasis criteria. Minor bleeding included subcutaneous ecchymosis, wound bleeding, and mild gastrointestinal bleeding, while major bleeding was defined as intracranial hemorrhage, transfusion requirement of ≥2 units of red blood cells, or bleeding leading to hemodynamic instability.

#### 2.4.3. Assessment of medication adherence

Adherence was comprehensively evaluated through structured questionnaires during follow-up, medical record review, and verification of medication use. Nonadherence was defined as failure to complete the prescribed anticoagulation course, including self-discontinuation, missed doses, or interruption due to financial or operational difficulties. Before discharge, all patients received detailed medication instructions, and during follow-up, self-reported adherence and caregiver feedback were documented by the study team.

#### 2.4.4. Assessment of postoperative recovery

Postoperative recovery outcomes included time to 1st ambulation and wound healing status, verified against nursing records and discharge summaries. Any cases of delayed healing or suspected wound infection were further recorded, along with corresponding management strategies and recovery progress.

### 2.5. Statistical analysis

All statistical analyses were performed using Statistical Package for the Social Sciences version 26.0 (IBM Corp., Chicago). Continuous variables were expressed as mean ± standard deviation ($\bar{x}\pm s$) and compared between groups using independent-sample *t* tests. Categorical variables were expressed as frequencies and percentages, with between-group comparisons conducted using the χ^2^ test or Fisher’s exact test, as appropriate. Cumulative survival rates were analyzed using the Kaplan–Meier method, and intergroup differences were assessed with the Log-rank test. A 2-tailed *P* value of <.05 was considered statistically significant.

## 3. Result

### 3.1. Baseline characteristics

A total of 100 elderly patients with hip fractures admitted to our hospital between January 2022 and January 2025 were enrolled in this study and assigned to the LMWH group (n = 50) or the rivaroxaban group (n = 50). The sample size was determined based on preliminary data, with a 2-sided α of 0.05 and a statistical power (1-β) of 0.80. An expected between-group difference in VTE incidence of approximately 6% was assumed. Sample size calculation using G*Power 3.1 software indicated that at least 45 patients per group were required. To ensure adequate statistical power and account for potential loss to follow-up, the final total sample size was set at 100 cases.

Baseline characteristics, including age, sex, height, weight, body mass index, blood pressure, heart rate, smoking history, surgical method, and common comorbidities, were compared between the 2 groups. No statistically significant differences were observed (*P* > .05), indicating good comparability between groups before treatment. Detailed results are presented in Table 1.

**Table 1:** Comparison of baseline characteristics between the 2 groups.

Baseline characteristics	LMWH group (n = 50)	Rivaroxaban group (n = 50)	*t*/χ^2^ value	*P* value
Age (yr)	79.4 ± 4.3	78.8 ± 5.1	*t* = 0.59	.56
Male (%)	40 (80%)	38 (76%)	χ^2^ = 0.14	.71
Height (cm)	162.4 ± 7.2	163.0 ± 6.9	*t* = 0.49	.63
Weight (kg)	65.3 ± 8.4	64.9 ± 7.9	*t* = 0.22	.83
BMI (kg/m^2^)	24.3 ± 3.2	24.1 ± 2.9	*t* = 0.37	.71
Systolic BP (mm Hg)	145.2 ± 15.1	144.8 ± 16.2	*t* = 0.13	.89
Diastolic BP (mm Hg)	85.3 ± 10.7	84.6 ± 11.2	*t* = 0.31	.76
Heart rate (beats/min)	76.2 ± 10.4	75.6 ± 9.7	*t* = 0.36	.72
Smoking history (%)	10 (20%)	12 (24%)	χ^2^ = 0.09	.76
Hypertension (%)	22 (44%)	23 (46%)	χ^2^ = 0.04	.86
Diabetes mellitus (%)	18 (36%)	17 (34%)	χ^2^ = 0.05	.82
Coronary heart disease (%)	8 (16%)	9 (18%)	χ^2^ = 0.06	.81
COPD (%)	6 (12%)	7 (14%)	χ^2^ = 0.08	.78
Surgical methods (hemiarthroplasty/total hip arthroplasty/PFNA fixation)	22/16/12	20/18/12	χ^2^ = 0.18	.91

BMI = body mass index, BP = blood pressure, COPD = chronic obstructive pulmonary disease, LMWH = low-molecular-weight heparin, PFNA = proximal femoral nail antirotation.

### 3.2. Incidence of DVT

**Figure 1.**
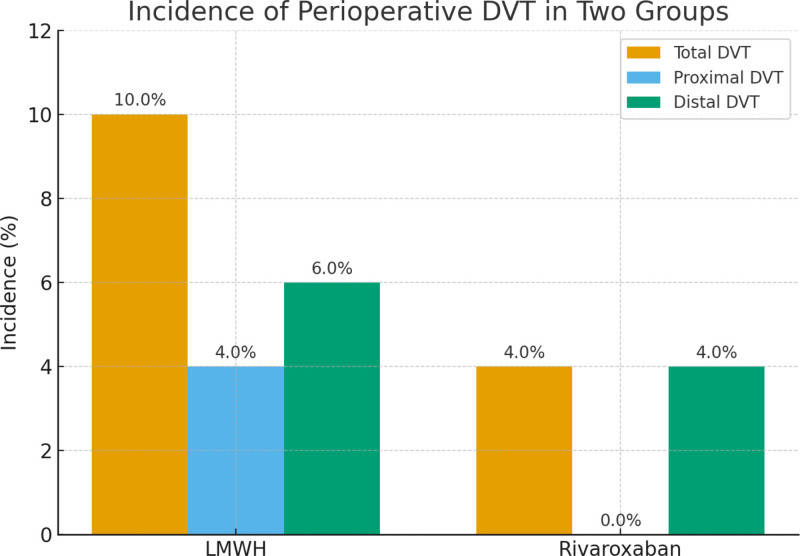
Incidence of perioperative DVT in 2 groups. DVT = deep vein thrombosis, LMWH = low-molecular-weight heparin.

A significant difference in perioperative DVT incidence was observed between the 2 groups. In the LMWH group, 5 patients (10.0%) developed DVT, including 2 cases of proximal DVT and 3 cases of distal DVT. In contrast, the rivaroxaban group reported only 2 cases (4.0%), both of which were distal DVT, with no proximal thrombosis detected. The overall incidence of DVT in the rivaroxaban group was significantly lower compared with the LMWH group (χ^2^ = 4.65, *P* = .03) (Table 2; Fig. 1).

**Table 2:** Comparison of perioperative DVT incidence between the 2 groups.

Variables	LMWH group (n = 50)	Rivaroxaban group (n = 50)	χ^2^/*t* value	*P* value
Overall DVT incidence	5 (10.0%)	2 (4.0%)	χ^2^ = 4.65	.03
Proximal DVT (%)	2 (4.0%)	0 (0%)	χ^2^ = 2.04	.15
Distal DVT (%)	3 (6.0%)	2 (4.0%)	χ^2^ = 0.21	.65
Time to onset (d)	8.6 ± 2.3	9.1 ± 1.7	*t* = 0.63	.53

DVT = deep vein thrombosis, LMWH = low-molecular-weight heparin.

**Figure 2.**
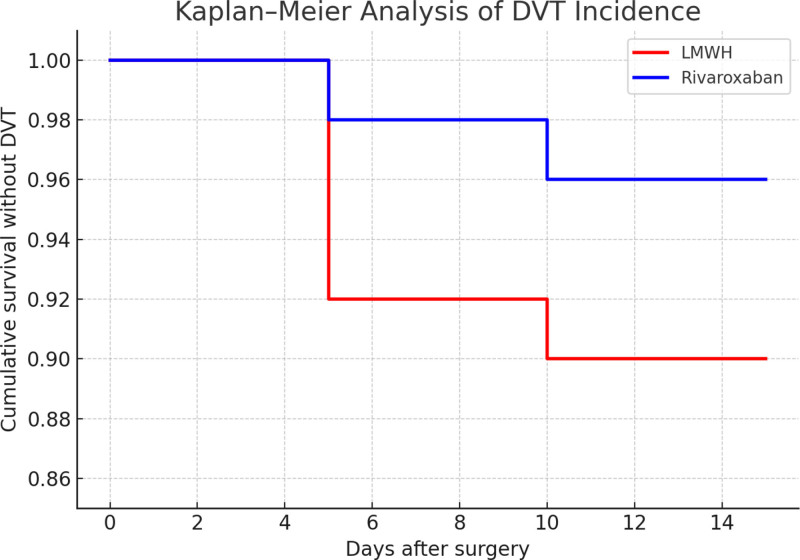
Kaplan–Meier analysis of DVT incidence. DVT = deep vein thrombosis.

Further analysis of the time to DVT onset showed that in the LMWH group, 4 cases occurred within 7 days postoperatively and 1 case within 14 days. In the rivaroxaban group, both cases developed within 10 days after surgery. Kaplan–Meier cumulative incidence curves demonstrated that rivaroxaban was associated with a higher perioperative thrombosis-free survival rate compared with LMWH, and the difference was statistically significant (log-rank χ^2^ = 4.38, *P* = .036) (Fig. 2).

These findings indicate that rivaroxaban provides superior protection in preventing perioperative DVT among elderly patients with hip fractures, with a lower incidence of thrombotic events compared to LMWH.

### 3.3. Incidence of bleeding complications

**Figure 3.**
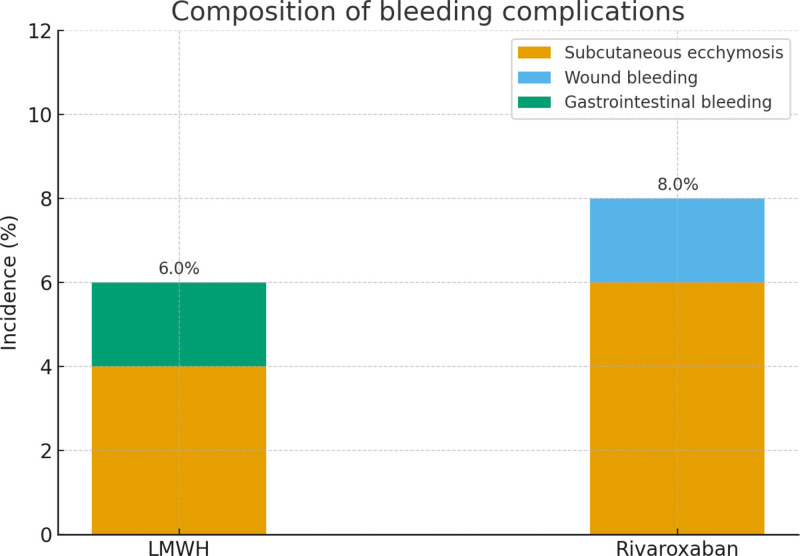
Composition of bleeding complications. LMWH = low-molecular-weight heparin.

Bleeding complications represent a critical indicator for evaluating the safety of anticoagulant therapy. In this study, 3 patients (6.0%) in the LMWH group developed bleeding events, including 2 cases of mild subcutaneous ecchymosis and 1 case of mild gastrointestinal bleeding. In the rivaroxaban group, 4 patients (8.0%) experienced bleeding events, consisting of 3 cases of mild subcutaneous ecchymosis and 1 case of wound oozing. The overall incidence of bleeding did not differ significantly between the 2 groups (χ^2^ = 0.10, *P* = .75) (Table 3; Fig. 3).

**Table 3:** Comparison of bleeding complications between the 2 groups.

Types of bleeding complications	LMWH group (n = 50)	Rivaroxaban group (n = 50)	χ^2^ value	*P* value
Overall bleeding events (%)	3 (6.0%)	4 (8.0%)	0.10	.75
Subcutaneous ecchymosis (%)	2 (4.0%)	3 (6.0%)	0.13	.72
Wound bleeding (%)	0 (0%)	1 (2.0%)	1.02	.31
Gastrointestinal bleeding (%)	1 (2.0%)	0 (0%)	1.02	.31
Severe bleeding (intracranial/required transfusion)	0 (0%)	0 (0%)	–	–

LMWH = low-molecular-weight heparin.

When analyzing specific types of bleeding, no cases of intracranial hemorrhage, fatal bleeding, or transfusion-requiring severe hemorrhage were observed in either group. The incidence of mild bleeding events was also comparable between the groups (χ^2^ = 0.13, *P* = .72). These findings suggest that rivaroxaban demonstrates a safety profile similar to LMWH when used in elderly patients with hip fractures during the perioperative period.

### 3.4. Mortality

During the perioperative period and follow-up, the overall incidence of mortality was relatively low. In the LMWH group, 1 patient (2.0%) died due to postoperative myocardial infarction. In the rivaroxaban group, 2 patients (4.0%) died – 1 from pulmonary infection and the other from multiple organ failure. The difference in mortality between the 2 groups was not statistically significant (χ^2^ = 0.16, *P* = .69).

**Figure 4.**
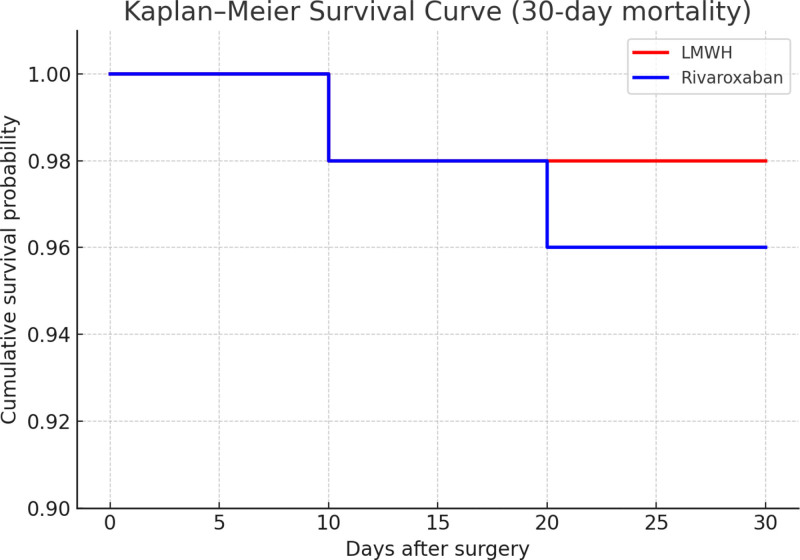
Kaplan–Meier survival curve (30-day mortality).

Further evaluation using Kaplan–Meier survival analysis demonstrated no significant difference in cumulative 30-day survival rates between the groups (log-rank χ^2^ = 0.21, *P* = .65). These results suggest that both LMWH and rivaroxaban provide comparable short-term survival outcomes when used for perioperative anticoagulation prophylaxis in elderly patients with hip fractures (Table 4; Fig. 4).

**Table 4:** Comparison of surgical and hospitalization parameters between the 2 groups.

Variables	LMWH group (n = 50)	Rivaroxaban group (n = 50)	χ^2^ value	*P* value
Overall mortality (%)	1 (2.0%)	2 (4.0%)	0.16	.69
Cardiovascular death (%)	1 (2.0%)	0 (0%)	1.02	.31
Infection/respiratory failure (%)	0 (0%)	1 (2.0%)	1.02	.31
Multiple organ failure (%)	0 (0%)	1 (2.0%)	1.02	.31
30-day cumulative survival (%)	98.0%	96.0%	0.21	.65

LMWH = low-molecular-weight heparin.

### 3.5. Medication adherence

During follow-up, differences in medication adherence were observed between the 2 groups. In the LMWH group, 3 patients (6.0%) failed to complete the recommended course of anticoagulation, primarily due to the inconvenience of self-administered subcutaneous injections, which most frequently occurred within 1 week after discharge. In contrast, only 1 patient (2.0%) in the rivaroxaban group discontinued treatment, citing financial burden as the reason. Overall, adherence was slightly better in the rivaroxaban group compared with the LMWH group, although the difference did not reach statistical significance (χ^2^ = 2.44, *P* = .12).

Analysis of reasons for nonadherence revealed that the inconvenience of injection was the predominant barrier in the LMWH group, whereas rivaroxaban oral administration clearly improved compliance. Nevertheless, economic constraints may still limit its broader application in some patients. Importantly, both groups achieved generally satisfactory adherence, and no serious thrombotic or bleeding complications were attributed to noncompliance.

### 3.6. Length of hospital stay and postoperative recovery

Significant differences were observed in postoperative recovery between the 2 groups. The mean length of hospital stay in the rivaroxaban group was 9.2 ± 2.3 days, which was significantly shorter than that in the LMWH group (10.1 ± 2.5 days; *t* = 2.08, *P* = .04). In addition, the mean time to 1st ambulation was 3.8 ± 1.2 days in the rivaroxaban group compared with 4.4 ± 1.3 days in the LMWH group, and this difference was also statistically significant (*t* = 2.12, *P* = .036). By contrast, no significant difference was found between the 2 groups in terms of wound healing time (*P* > .05) (Table 5).

**Table 5:** Comparison of surgical and hospitalization parameters between the 2 groups.

Variables	LMWH group (n = 50)	Rivaroxaban group (n = 50)	*t* value	*P* value
Length of hospital stay (d)	10.1 ± 2.5	9.2 ± 2.3	2.08	.04
Time to 1st ambulation (d)	4.4 ± 1.3	3.8 ± 1.2	2.12	.036
Wound healing time (d)	12.5 ± 3.1	12.2 ± 2.9	0.49	.62

LMWH = low-molecular-weight heparin.

Overall, patients in the rivaroxaban group demonstrated faster postoperative recovery and shorter hospital stays, which may be attributed to the convenience of oral administration, improved adherence, and reduced dependence on nursing care.

## 4. Discussion

Hip fractures are among the most common orthopedic emergencies in the elderly, with a rising incidence due to global population aging.^[25,26]^ It is associated with high rates of disability, loss of function, and mortality, posing a substantial challenge to both patient quality of life and healthcare systems. Elderly patients typically present with multiple comorbidities and diminished physiological reserves, placing them at elevated risk of perioperative complications. Among these, VTE – including DVT and pulmonary embolism – remains a leading cause of morbidity and mortality. Thus, achieving an optimal balance between effective thromboprophylaxis and avoidance of bleeding risk is central to perioperative anticoagulation management in this population. LMWH has long been regarded as the standard prophylactic agent, with well-established efficacy and safety. However, its injectable administration often impairs adherence, particularly in frail elderly patients. Rivaroxaban, an oral factor Xa inhibitor, offers the advantages of oral dosing and predictable pharmacokinetics, yet evidence in elderly hip fracture populations remains limited.^[27,28]^ The present study aimed to address this gap by comparing rivaroxaban with LMWH in terms of efficacy, safety, adherence, and recovery outcomes.

This study demonstrated comparable baseline characteristics between groups, confirming the validity of outcome comparisons. In terms of efficacy, rivaroxaban significantly reduced the incidence of perioperative DVT compared with LMWH, with Kaplan–Meier analysis further supporting superior thrombus-free survival. These findings are consistent with prior literature, yet differ from most existing studies that primarily focused on elective arthroplasty patients.^[29,30]^ By targeting elderly hip fracture patients – a group with higher perioperative vulnerability – this study provides more clinically relevant evidence and expands current knowledge in this underexplored domain.

Safety outcomes further support rivaroxaban as a feasible alternative. The incidence of bleeding complications was similar between groups, with no cases of severe bleeding or transfusion-requiring events. These results suggest that rivaroxaban does not increase hemorrhagic risk in elderly hip fracture patients, aligning with prior findings in other orthopedic populations. Importantly, given the heightened bleeding susceptibility in older patients with renal or hepatic impairment and polypharmacy, safety data specific to this subgroup are particularly valuable.

When extending safety assessment to mortality, both groups demonstrated low 30-day mortality rates with no significant differences. Compared with previously reported mortality rates of 5% to 10% in elderly hip fracture patients,^[31,32]^ the overall mortality in this study was relatively low, likely reflecting standardized surgical care and perioperative management in our cohort. This suggests that while anticoagulant selection may not markedly impact short-term mortality, it remains crucial for reducing VTE events and improving postoperative quality of life.

Adherence emerged as another key factor influencing treatment effectiveness. The rivaroxaban group demonstrated slightly better adherence than the LMWH group, although the difference did not reach statistical significance. The primary barrier for LMWH was injection inconvenience, frequently leading to discontinuation after discharge, whereas rivaroxaban oral administration enhanced patient convenience. Nonetheless, economic burden contributed to treatment discontinuation in some rivaroxaban patients, highlighting the importance of considering pharmacoeconomic factors when implementing prophylaxis strategies in the elderly population.

Regarding postoperative recovery, rivaroxaban was associated with shorter hospital stays and earlier ambulation, while wound healing times were similar across groups. Improved recovery may be attributed to better adherence, reduced nursing dependence, and the simplicity of oral administration. As previous studies have emphasized, shorter hospitalization not only decreases healthcare costs but also reduces nosocomial infection risks.^[33]^ Our results reinforce this notion, adding further evidence in support of rivaroxaban’s broader clinical benefits.

The strengths of this study include its focused elderly population and comprehensive outcome evaluation beyond thrombosis and bleeding. These factors enhance the reliability and clinical applicability of the findings. Nevertheless, certain limitations should be acknowledged. First, this was a single-center, retrospective study, with potential for selection bias. Second, the follow-up period was relatively short, limiting assessment of long-term outcomes. Third, although the sample size was adequately powered, larger multicenter trials are required to validate these results. Finally, pharmacoeconomic analysis was not included, an important consideration for elderly patients requiring prolonged therapy. Although all patients underwent ultrasound examinations following the same institutional protocol, fixed-point screening (postoperative days 7 and 14, and at discharge) may have failed to capture early or late thrombotic events, introducing potential detection bias. Furthermore, this study did not apply statistical adjustments for multiple comparisons, which may increase the risk of type I errors when interpreting secondary outcomes. In addition, although the baseline characteristics between groups were generally comparable, no multivariable regression analysis was performed to control for potential confounding factors, such as comorbidities, functional status, or surgical type. Future prospective studies with more robust statistical modeling are needed to validate the independent associations and strengthen causal inferences.

In conclusion, rivaroxaban demonstrated superior efficacy in preventing perioperative DVT in elderly hip fracture patients compared with LMWH, with comparable safety, slightly better adherence, and advantages in postoperative recovery. These findings suggest rivaroxaban may represent a preferred option for anticoagulation in this high-risk population. However, future large-scale, prospective, multicenter studies with longer follow-up are warranted to confirm its clinical utility and cost-effectiveness.

## 5. Conclusion

This study systematically compared the efficacy of LMWH and rivaroxaban for perioperative thromboprophylaxis in elderly patients with hip fractures. The findings demonstrated that rivaroxaban was superior to LMWH in reducing the incidence of DVT without increasing the risk of bleeding. Both groups maintained relatively low short-term mortality under standardized perioperative management, suggesting that rivaroxaban offers favorable efficacy and safety in this high-risk population. Moreover, rivaroxaban showed slightly better treatment adherence due to its convenient oral administration, and it was associated with shorter hospitalization and earlier postoperative mobilization, reflecting potential advantages in recovery and healthcare efficiency. These results highlight not only the feasibility of rivaroxaban in clinical practice but also its added value in improving patient experience and optimizing perioperative management.

The novelty of this study lies in its focus on an elderly, high-risk population and in the multidimensional evaluation of treatment outcomes, including efficacy, safety, adherence, and recovery. Such a comprehensive perspective enhances the clinical relevance of the conclusions. Nevertheless, the limitations of a single-center, retrospective design, limited sample size, and relatively short follow-up period may restrict the generalizability of the findings. Future prospective, multi-center studies with larger cohorts and longer follow-up, as well as pharmacoeconomic analyses, are warranted to validate and extend these conclusions. Overall, rivaroxaban appears to be a promising alternative to LMWH for perioperative thromboprophylaxis in elderly patients with hip fractures, providing a valuable reference for clinical decision-making.

## Author contributions

**Conceptualization:** Xueqin He, Hui Chen, Fan Lou, Chen Yan, Jing Feng.

**Funding acquisition:** Xueqin He, Hui Chen.

**Data curation:** Xueqin He, Hui Chen, Fan Lou, Chen Yan, Jing Feng.

**Formal analysis:** Xueqin He, Hui Chen, Fan Lou, Chen Yan, Jing Feng.

**Investigation:** Xueqin He.

**Writing – original draft:** Xueqin He, Fan Lou, Chen Yan, Jing Feng.

**Writing – review & editing:** Xueqin He, Fan Lou, Chen Yan, Jing Feng.
